# An *MSC2* Promoter-*lacZ* Fusion Gene Reveals Zinc-Responsive Changes in Sites of Transcription Initiation That Occur across the Yeast Genome

**DOI:** 10.1371/journal.pone.0163256

**Published:** 2016-09-22

**Authors:** Yi-Hsuan Wu, Janet Taggart, Pamela Xiyao Song, Colin MacDiarmid, David J. Eide

**Affiliations:** Department of Nutritional Sciences, University of Wisconsin Madison, Madison, Wisconsin, United States of America; Southern Illinois University School of Medicine, UNITED STATES

## Abstract

The Msc2 and Zrg17 proteins of *Saccharomyces cerevisiae* form a complex to transport zinc into the endoplasmic reticulum. *ZRG17* is transcriptionally induced in zinc-limited cells by the Zap1 transcription factor. In this report, we show that *MSC2* mRNA also increases (~1.5 fold) in zinc-limited cells. The *MSC2* gene has two in-frame ATG codons at its 5’ end, ATG1 and ATG2; ATG2 is the predicted initiation codon. When the *MSC2* promoter was fused at ATG2 to the *lacZ* gene, we found that unlike the chromosomal gene this reporter showed a 4-fold decrease in *lacZ* mRNA in zinc-limited cells. Surprisingly, β-galactosidase activity generated by this fusion gene increased ~7 fold during zinc deficiency suggesting the influence of post-transcriptional factors. Transcription of *MSC2*^*ATG2*^*-lacZ* was found to start upstream of ATG1 in zinc-replete cells. In zinc-limited cells, transcription initiation shifted to sites just upstream of ATG2. From the results of mutational and polysome profile analyses, we propose the following explanation for these effects. In zinc-replete cells, *MSC2*^*ATG2*^*-lacZ* mRNA with long 5’ UTRs fold into secondary structures that inhibit translation. In zinc-limited cells, transcripts with shorter unstructured 5’ UTRs are generated that are more efficiently translated. Surprisingly, chromosomal *MSC2* did not show start site shifts in response to zinc status and only shorter 5’ UTRs were observed. However, the shifts that occur in the *MSC2*^*ATG2*^*-lacZ* construct led us to identify significant transcription start site changes affecting the expression of ~3% of all genes. Therefore, zinc status can profoundly alter transcription initiation across the yeast genome.

## Introduction

Zinc is an essential nutrient for function of the secretory pathway by serving as a cofactor for both secreted proteins and proteins that reside in secretory pathway compartments. The importance of regulatory mechanisms for maintaining zinc homeostasis in the secretory pathway of *Saccharomyces cerevisiae* was recently demonstrated by studies of Zrg17, a zinc transporter in the endoplasmic reticulum (ER) that supplies zinc to that organelle. *ZRG17* gene expression is induced in zinc-limited cells and this gene is a direct target gene of the Zap1 transcription factor [1]. In yeast, Zap1 is the central player in zinc homeostasis where it activates the expression of ~80 genes in response to zinc deficiency [2]. Disruption of Zap1-mediated induction of *ZRG17* under zinc-limited conditions led to elevated ER stress, thus demonstrating the biological importance of this regulation to maintaining ER function.

Zrg17 does not function as a zinc transporter by itself. Previous results indicated that zinc transport into the ER is active only when Zrg17 forms a heteromeric complex with the Msc2 protein [3]. Both Msc2 and Zrg17 are members of the cation diffusion facilitator family of metal transporters [4] and are orthologous to the vertebrate ZnT5 and ZnT6 proteins, respectively [5]. Although *ZRG17* was shown to be regulated by Zap1, genome-wide microarray analyses failed to identify changes in *MSC2* mRNA levels in response to zinc status [6,7]. Therefore, in this report, we set out to characterize the regulation of the *MSC2* gene. While microarray analyses did not detect changes in *MSC2* expression, the more sensitive methods of S1 nuclease protection assay and quantitative RT-PCR independently showed that there was a small but reproducible increase in *MSC2* mRNA level in zinc-limited vs. zinc-replete cells. When we fused a 500 bp fragment of the *MSC2* promoter to *lacZ*, we found that mRNA from this reporter decreased 4-fold in zinc-limited cells, indicating that the promoter fragment used was insufficient to confer wild-type *MSC2* regulation on the reporter gene. Surprisingly, however, β-galactosidase enzyme activity generated by the reporter was highly induced in zinc-limited cells despite the decrease in mRNA level. Transcription start site mapping showed that this induction correlated with a shift in the site of transcription initiation in response to zinc status. Transcripts with longer 5’UTRs produced in zinc-replete cells appear to be poorly translated while shorter transcripts produced in zinc-limited cells are translated more efficiently.

Transcription start site selection is a complex process requiring a number of different basal cofactors [8]. Following assembly of the preinitiation complex on a promoter, the TFIIH helicase unwinds the DNA strands adjacent to the transcription start site to form the “open complex” conformation. RNA polymerase II then scans downstream for a suitable transcription start site. Previous studies have shown that TFIIB, TFIIF and Pol II are all involved in start site selection because mutations in these factors have been found to alter where transcription initiation occurs [9–12]. Notably, several subunits of these factors are zinc-dependent so zinc status could potentially have an impact on transcription start sites of other yeast genes through this or other zinc-related mechanisms. Consistent with this hypothesis, we found that genes throughout the genome have altered transcription start sites in zinc deficiency. Thus, the artificial *MSC2-lacZ* reporter fusion led us to find that zinc status alters transcription start site selection at many genes in yeast.

## Materials and Methods

### Yeast Strains and Growth Conditions

Media used were YPD, SD, and LZM as described previously [13]. LZM contains 1 mM EDTA and 20 mM citrate to both limit and buffer available zinc levels. In all experiments, 2% glucose was used as the carbon source. Zinc was supplied as ZnCl_2_. Yeast strains used were wild type DY150 (*MATa ade2 can1 his3 leu2 trp1 ura3)*, *msc2Δ* (DY150 *msc2Δ*::*HIS3*) [14], ABY9 (*MATα ade6 can1 his3 leu2 trp1 zap1Δ*::*KanMX4)* [15], BY4743 (*MATa/MATα his3Δ1/his3Δ1 leu2Δ0/leu2Δ0 lysΔ0/LYS2 MET15/met15Δ0 ura3Δ0/ura3Δ0*) (Invitrogen), BY4743 *rpd9Δ* (Invitrogen), H51 (*MATa his3Δ200 ura3-52*, *leu2-3*, *112*), YMH650 (H51 *ssu72-2*), YMH14 (*MATa cyc1-5000 cyc7-67 cyh2 leu3-3*,*112 ura3-52*), YMH124 (YMH14 *sua7-1*), and YDW383 (YMH14 *sua8-1*). The YMH strains and YDW383 were all provided by Michael Hampsey (Rutgers University).

### Yeast Plasmids

The reporter plasmid *MSC2*^ATG2^*-lacZ* was constructed in *lacZ* fusion vector YEp353 by homologous recombination. PCR products were amplified from pMSC2 [16] that contained 500 bp of *MSC2* promoter sequence and the ATG2 initiation codon (see below) flanked by sequences homologous to the vector. This fragment was gel purified and co-transformed with EcoRI- and PstI-digested YEp353 [17]; transformants were selected for *URA3* prototrophy. Reporter plasmid *MSC2*^ATG1^*-lacZ* was constructed in the same way as *MSC2*^ATG2^*-lacZ* but used ATG1 as the initiation codon. The ATG mutants (*MSC2*^ATG2^*-lacZ* mATG1, *MSC2*^ATG2^*-lacZ* mATG2) were constructed in a similar fashion after generating the point mutation fragments by overlapping PCR. All plasmids were confirmed by DNA sequencing.

### RNA Analyses

S1 nuclease protection assays were performed with total RNA as previously described [18]. Total RNA was extracted from cells grown to mid-log phase with hot acid phenol. For each reaction, 15 μg of total RNA was hybridized to ^32^P-end-labeled DNA oligonucleotide probes for *MSC2*, *lacZ*, and *CMD1* before digestion with S1 nuclease and separation on 10% polyacrylamide, 5 M urea polyacrylamide gels. The gels were subsequently dried and exposed to phosphor screens. Band intensities were imaged by Typhoon FLA 7000 (GE Healthcare Life Sciences) and quantified using ImageQuant TL analysis software (GE Healthcare Life Sciences). Quantitative RT-PCR analysis was performed as previously described [19]. Additional primer sequences used were (5'-3'): GGCCGCATATGCTCTCTTA and ACTTATCGAATGCTGTGCGA for *MSC2*, and AACGACATTGGCGTAAGTGA and CCGCATCAGCAAGTGTATCT for *lacZ*.

### β-galactosidase Assays

β-galactosidase activity was measured using the Beta-Glo assay system (Promega). Cells were grown to mid-log phase in LZM supplemented with the indicated amount of ZnCl_2_. After cells were diluted to an OD_600_ of 0.1 (~2 x 10^6^ cells/ml), an equal volume of Beta-Glo reagent was then added and the mixture was incubated at room temperature for 60 min before assaying in a Turner 20/20n luminometer (Promega). β-galactosidase activity is reported in relative light units (x 10^5^ for *MSC2-lacZ* reporters and x 10^7^ for *HIS4-lacZ*).

### 5’ RLM-RACE

To map 5’ transcription start sites of ^7m^G-capped mRNA, 5’ RNA Ligase Mediated-Rapid Amplification of cDNA Ends (5’ RLM-RACE) was performed using the FirstChoice® RLM-RACE Kit (Invitrogen). Total RNA was extracted from wild-type (DY150) cells expressing the *MSC2*^ATG2^*-lacZ* reporter with hot acid phenol, and used as a template for 5’ RLM-RACE. The primers used in the outer PCR were 5’ RACE outer primer (5'-GCTGATGGCGATGAATGAACACTG-3') and outer *lacZ*-specific primer (5’-TGGGATAGGTTACGTTGGTGT-3’) or outer *MSC2*-specific primer (5’- GGTTAAAGGAAGAAGCTACGAAGG-3’). The primers used for the inner PCR were 5’ RACE inner primer (5'-CGCGGATCCGAACACTGCGTTTGCTGGCTTTGATG-3') and inner *lacZ*-specific primer (5’-CGCGAATTCCAAGGCGATTAAGTTGGGTAAC-3’) or inner *MSC2*-specific primer (5’-CGCGAATTCGGAAGGTACGATCAGATTACTGG-3’).

### Cloning of 5’ RLM-RACE PCR Products

The gel-purified RLM-RACE PCR products were ligated into a pBluescript SK (+) (Stratagene) that had been double-digested with BamHI (Promega) and EcoRI (Promega) restriction endonucleases and transformed into *E*. *coli*. The 5' RACE inner primer has a BamH1 site at its 5' end while the inner *lacZ*-specific primer and inner *MSC2* primer has EcoRI sites at their 5’ ends. Plasmid constructs were confirmed by DNA sequencing.

### Mfold Analysis of RNA Structure

The 5’ *MSC2*^ATG2^*-lacZ* long transcript RNA was analyzed with the Mfold web server (http://unafold.rna.albany.edu/?q=mfold), which predicts RNA secondary structures based on the free energy minimization method [20].

### Polysome Profiling on Sucrose Gradients

Wild-type (DY150) cells expressing *MSC2*^ATG2^*-lacZ* reporter were grown to mid-log phase, treated with cycloheximide (100 μg/ml), and then immediately transferred to an ice water bath. Cells were harvested by centrifugation at 4°C and washed in 2.5 ml polysome lysis buffer (20 mM Tris-HCl [pH 8.0], 140 mM KCl, 1.5 mM MgCl_2_, 0.5 mM dithiothreitol, 1% Triton X-100, 100 μg /ml cycloheximide, 0.1 mg/ml heparin). Cells were subsequently resuspended in ≈ 700 μl polysome lysis buffer and lysed in 2.0 ml microfuge tubes by vortexing for 5 min at 4°C in the presence of chilled glass beads. After two rounds of centrifugation at 4°C (500 x g, 5 minutes), the resulting lysates (~500 μl) were layered on 15–60% linear sucrose gradients. Gradients were centrifuged at 39,000 rpm for 2 h at 4°C in a Beckman SW 40Ti rotor and then fractionated using an Isco Model UA-6 gradient fractionator. The absorbance at 254 nm was continuously monitored and 1-ml fractions were collected. RNA was extracted from each collected fraction with RNA STAT-60 reagent (Tel-Test, Inc.). S1 nuclease protection assays were performed as described above to assess *MSC2*, *lacZ*, and *CMD1* RNA distribution in the fractions collected.

### Mapping of transcription start sites by 5’ Deep-RACE

Transcription start sites were mapped at the whole genome level using the 5’ Deep-RACE method [21]. In 5’ Deep-RACE, the gene-specific 5’ RLM-RACE method is adapted to a genome-wide analysis of mRNA 5’ ends. Wild-type (DY150) cells were grown in zinc-replete (LZM + 100 μM ZnCl_2_) and zinc-deficient (LZM + 1 μM ZnCl_2_) media (six replicates each). Total RNA was isolated from each culture and equal amounts of RNA from the replicate samples were pooled. Using the FirstChoice® RLM-RACE kit (Ambion), the RNA samples were treated first with Calf Intestinal Alkaline Phosphatase, then with Tobacco Acid Pyrophosphatase followed by ligation with the kit’s 5’RACE RNA adaptor. M-MLV reverse transcriptase was used to make cDNA using a random primer oligonucleotide (5’-CACGAGCGGTGACTGGAGTTCAGACGTGTGCTCTTCCGATCTNNNNNN-3’ where N = random nucleotides), and the cDNA products were then PCR-amplified using nested primer sets (1^st^ round outer primers, 10 cycles): 5’-GCTGTGGCGATGAATACAC-3’, 5’-CACGAGCGGTGACTGGAGTTC-3’; 2^nd^ round inner primers (15 cycles): 5’-ACACTCTTTCCCTACACGAC-3’, 5’-GTGACTGGAGTTCAGACGTGTGC-3’) to generate a library of fragments containing the 5’ends of mRNA. These fragments were sequenced by DNA sequencing on an Illumina HiSeq2000 machine using 1x 100-bp reads. Sequence image analysis and base calling were performed using the CASAVA 1.7.0 pipeline (Illumina). Primary analysis of the data (mapping and trimming) was performed with CLC Genomics Workbench 4.7.1 and mapped to the reference S288c sequence build (as of June 2011). Six million (zinc deficient) and 8 million (zinc replete) independent sequencing reads representing mRNA 5’ ends were obtained.

## Results

### Regulation of the *MSC2* promoter by zinc

**Fig 1:**
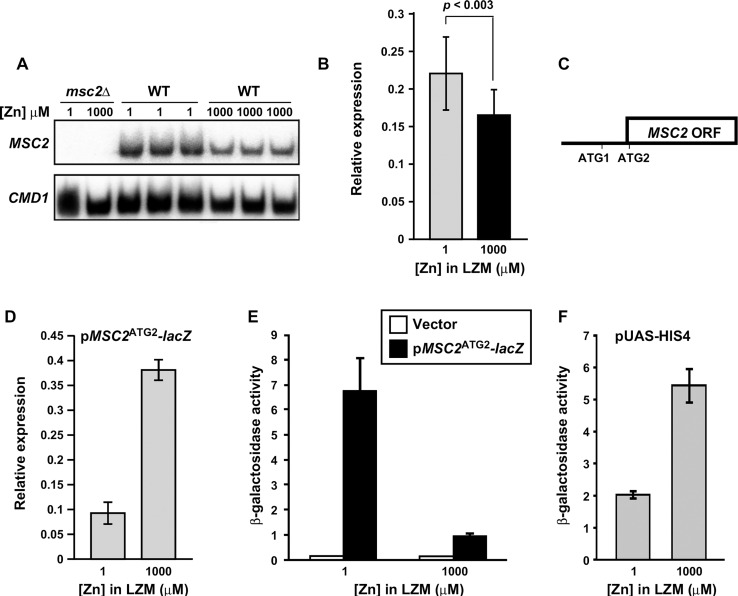
Effects of zinc status on chromosomal *MSC2* and plasmid *MSC2*^*ATG2*^*-lacZ* expression. A) *MSC2* mRNA levels were measured by S1 nuclease protection assay of RNA isolated from *msc2****Δ*** mutant (DY150 *msc2****Δ***) and wild-type (DY150) cells grown under zinc-limiting (LZM + 1 μM ZnCl_2_) or replete (LZM + 1000 μM ZnCl_2_) conditions. *CMD1* was used as a loading control. B) The mRNA abundance of *MSC2* in zinc-limited and replete DY150 cells was also determined by quantitative RT-PCR. *MSC2* abundance was normalized to the average abundance of three control transcripts (18S rRNA, *TAF10*, and *ACT1*). The data plotted represent the means of fifteen replicates from each condition and the error bars denote ± 1 S.D (p <0.003 as determined by the Student’s paired t-test). C) Diagram of *MSC2* with two in-frame ATGs at the 5’ end of the open reading frame indicated. ATG2 is the predicted translation start site, ATG1 is located 48 nucleotides upstream of ATG2, and the next in-frame ATG (ATG3) in the ORF is ~700 bp downstream of ATG2. Several out-of-frame ATGs are found in the interval ATG2 and ATG3. D) *lacZ* mRNA levels were measured by quantitative RT-PCR using RNA isolated from wild-type (DY150) cells transformed with the *MSC2*^ATG2^*-lacZ* reporter and grown in LZM supplemented with the indicated concentration of ZnCl_2_ as in panel B. The data plotted represent the means of three replicates from each condition and the error bars denote ± 1 S.D. Panels E, F) β-galactosidase activity was measured in wild-type (DY150) cells bearing the *lacZ* vector (YEp353), the *MSC2*^ATG2^*-lacZ* reporter, or a *HIS4-lacZ* fusion (pUAS-HIS4) grown under zinc-limiting (LZM + 1 μM ZnCl_2_) or replete (LZM + 1000 μM ZnCl_2_) conditions. Results are the means ± S.D. for three independent cultures for each condition and are representative of two independent experiments.

The results of our previous genome-wide microarray analyses suggested that *MSC2* mRNA levels are not greatly affected by zinc status [6,7]. To assess this question using a more sensitive method, we measured *MSC2* mRNA levels in cells grown under zinc-limiting (LZM + 1 μM ZnCl_2_) and replete (LZM + 1000 μM ZnCl_2_) conditions by S1 nuclease protection assay. As shown in **Fig 1A**, *MSC2* mRNA in wild-type cells was reproducibly more abundant in zinc-limited cells than in replete cells. After normalization using *CMD1* calmodulin mRNA levels, we determined that *MSC2* mRNA levels increased ~1.5 fold (1.46 ± 0.11 standard deviation) in zinc-limited cells. This induction was confirmed by quantitative RT-PCR (1.4-fold, **Fig 1B**) (p < 0.003). These data indicate that *MSC2* expression is regulated to a small but detectable degree in response to zinc status.

To determine whether this effect was due to zinc-responsive changes in the activity of the *MSC2* promoter, we fused that promoter to the *E*. *coli lacZ* gene. The *MSC2* open reading frame has two in-frame ATG codons near its 5’ end that we designated ATG1 and ATG2. ATG2 is located 48 nucleotides downstream of ATG1 (**Fig 1C**). Because ATG1 of the *S*. *cerevisiae MSC2* gene is not conserved among closely related *Saccharomyces* species, and the third in-frame ATG codon is 708 bp farther downstream in the *MSC2* coding sequence, ATG2 was deemed to be the likely initiation codon of this open reading frame. A 500 bp fragment containing the *MSC2* promoter was fused at ATG2 to *lacZ* to generate the *MSC2*^*ATG2*^*-lacZ* reporter gene fusion. The *MSC2* gene cloned in a low copy plasmid vector with the same 500 bp promoter fragment fully complemented the growth defect of an *msc2* mutant in zinc-limited medium suggesting that the gene’s full promoter is contained within this fragment [16]. However, when *lacZ* mRNA generated by the *MSC2*^*ATG2*^*-lacZ* plasmid was measured in zinc-limited vs. zinc-replete cells, we found that expression decreased 4-fold in low zinc (**Fig 1D**). This result suggests that the promoter fragment used to generate the *lacZ* reporter is not sufficient to confer normal regulation of the *MSC2* gene. Surprisingly, when *MSC2*^*ATG2*^*-lacZ* expressing cells were assayed for β-galactosidase activity, we found an opposite effect of zinc status; β-galactosidase activity increased ~7 fold in zinc-limited vs. replete cells (**Fig 1E**) despite the decrease in mRNA levels. Cells expressing a *HIS4-lacZ* fusion (pUAS-HIS4) [22] served as a control to show that elevated zinc levels do not generally inhibit β-galactosidase activity (**Fig 1F**). In fact, β-galactosidase activity from this reporter was ~3-fold higher in zinc-replete cells.

### Zinc-responsive *MSC2*^ATG2^*-lacZ* expression is Zap1-independent, zinc-specific, and not due to altered protein stability

**Fig 2:**
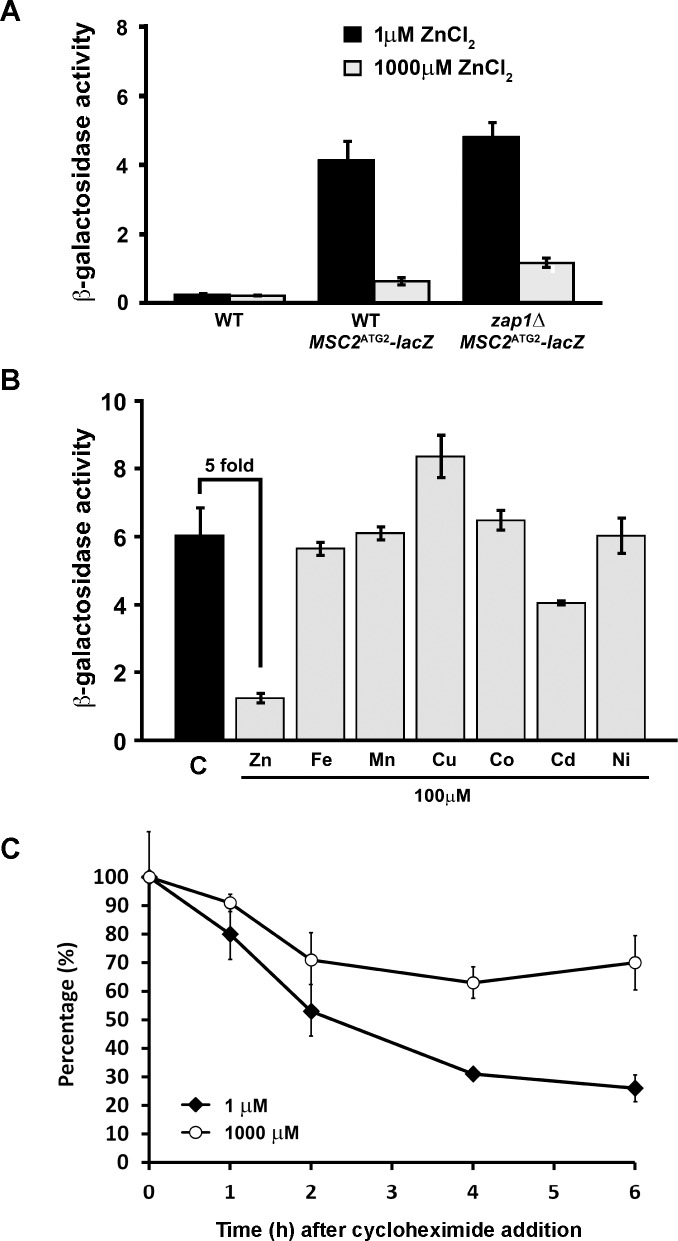
Zinc-responsive *MSC2*^ATG2^-*lacZ* activity is Zap1-independent, zinc-specific, and not due to differential degradation. A) β-galactosidase activity was measured in wild-type (DY150) or *zap1Δ* mutant (ABY9) cells expressing the *MSC2*^ATG2^*-lacZ* reporter and grown under zinc-limiting (LZM + 1 μM ZnCl_2_) or replete (LZM + 1000 μM ZnCl_2_) conditions. Activity measured in untransformed wild-type cells was used as negative control. B) β-galactosidase activity was measured in wild-type (DY150) cells expressing the *MSC2*^ATG2^*-lacZ* reporter. The cells were grown under zinc-limiting conditions (LZM + 1 μM ZnCl_2_) either without (control, C) or with addition of 100 μM of zinc (ZnCl_2_), iron (FeCl_3_), manganese (MnCl_2_), copper (CuCl_2_), cobalt (CoCl_2_), cadmium (CdCl_2_), or nickel (NiCl_2_). C) β-galactosidase protein is less stable in zinc-limited cells than in replete cells. β-galactosidase activity was measured in wild-type (DY150) cells transformed with *MSC2*^ATG2^*-lacZ* reporter and grown under zinc-limiting (LZM + 1 μM ZnCl_2_) or replete (LZM + 1000 μM ZnCl_2_) conditions. Cycloheximide (100 μg/ml) was added to the cells at time 0 and culture aliquots were removed at the indicated times for β-galactosidase activity measurements. Data are plotted as the percentage of the activity measured at time 0. For all panels, the results plotted are the means ± S.D. for three independent cultures for each condition and are representative of two independent experiments.

Comparison of the results shown in Fig 1D and 1E suggested that the expression of *MSC2*^*ATG2*^*-lacZ* was influenced by factors unrelated to changes in mRNA levels. To explore the mechanism(s) involved, we first determined whether Zap1, the major zinc-responsive transcriptional regulator in yeast was required for the effect. As shown in **Fig 2A**, deletion of the *ZAP1* gene had little effect on zinc-responsive changes in β-galactosidase activity generated by *MSC2*^*ATG2*^*-lacZ* indicating that the affected process is Zap1-independent. To determine if the effect was specific to zinc vs. other metals, zinc-limited cells expressing *MSC2*^*ATG2*^*-lacZ* were grown in zinc-limiting media alone or zinc-limiting media supplemented with 100 μM Zn, Fe, Mn, Cu, Co, Cd, and Ni (**Fig 2B**). Only the addition of zinc and, to a lesser extent cadmium, reduced β-galactosidase activity. Thus, the response of *MSC2*^*ATG2*^*-lacZ* is relatively zinc-specific.

The observation that **β**-galactosidase activity generated from *MSC2*^ATG2^*-lacZ* was highly induced by zinc limitation despite a decrease in *lacZ* mRNA could be explained by two possible mechanisms: differences in protein degradation or translational control. If differential degradation was responsible, the rate of lacZ protein degradation would be higher in zinc-replete cells and lower in limited cells thereby allowing the protein to accumulate to higher levels under zinc deficiency. To test this hypothesis, we used a cycloheximide chase method. Wild-type cells expressing *MSC2*^*ATG2*^*-lacZ* were grown in either zinc-limited or replete media. At time 0, cycloheximide was added to block additional protein synthesis and the stability of the pre-existing pool of lacZ protein was determined by β-galactosidase assay of samples harvested over a 6-hour period. As shown in **Fig 2C**, loss of β-galactosidase activity was faster in zinc-limited cells rather than slower as the hypothesis predicted. Thus, differential protein degradation does not explain the elevated levels of lacZ activity detected under zinc limitation.

**Fig 3:**
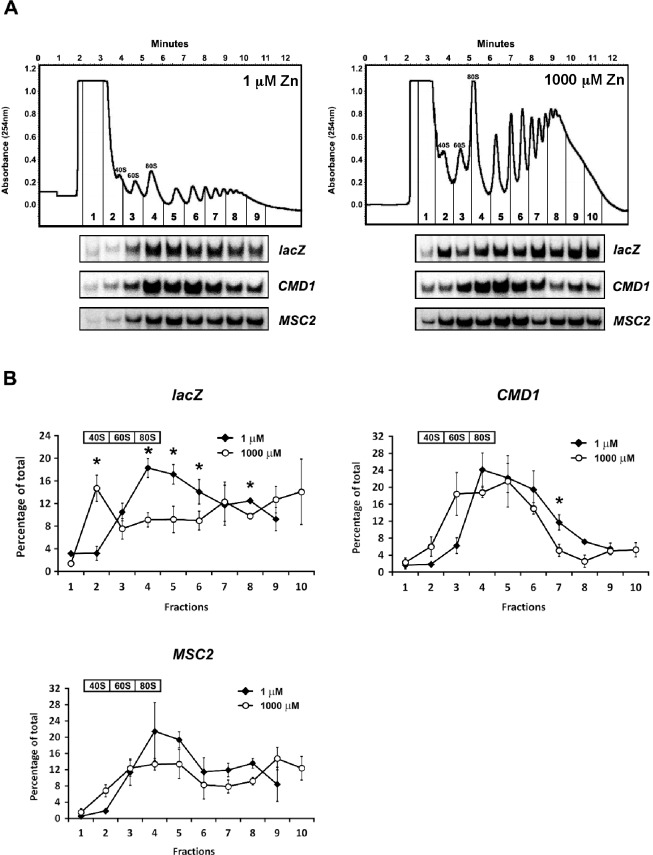
Polysome profile analysis of *MSC2*^*ATG2*^*-lacZ* mRNA. A) Representative polysome profiles of wild-type (DY150) cells expressing the *MSC2*^*ATG2*^*-lacZ* reporter and grown under zinc-limiting (LZM + 1 μM ZnCl_2_) or replete (LZM + 1000 μM ZnCl_2_) conditions. The positions of the 40S, 60S, 80S peaks are indicated and the collected fractions are numbered. RNA was extracted from each fraction and S1 nuclease protection assays were performed to detect *lacZ*, *CMD1*, and chromosomal *MSC2* mRNA. B) Quantified *lacZ*, *CMD1*, and *MSC2* mRNA abundance were plotted as the percentage of their total amount in all fractions. Results are the means ± S.D. for three independent cultures for each condition. The fractions containing the 40S, 60S, and 80S peaks are indicated and the *asterisks* indicate significant differences (*p* < 0.05) between zinc-replete and zinc-limited samples as determined by the paired Student’s t-test.

To assess whether zinc status affects translation of *MSC2*^*ATG2*^*-lacZ* mRNA, we performed a polysome profile analysis. Cells expressing this reporter gene were grown in zinc-limiting and replete conditions, lysed, and free 40S and 60S subunits, 80S ribosomes, and polyribosomes were separated by sucrose gradient fractionation. Despite preparing samples from similar numbers of cells, the 40S, 60S, 80S and polysome peaks in zinc-limited cells were much smaller than those from zinc-replete samples indicating that total ribosome number is lower in zinc-limited cells (**Fig 3A**). This result is consistent with the slower growth rate of these cells and reflects the normal regulation of ribosome biogenesis in response to growth [23]. The resulting gradient fractions were isolated and the distributions of *MSC2*^*ATG2*^*-lacZ* and *CMD1* mRNA were determined by S1 nuclease protection assay (**Fig 3A**). The mRNA levels in triplicate samples for each condition were assayed and the quantified data are plotted in **Fig 3B**. The *MSC2*^*ATG2*^*-lacZ* mRNA showed a marked transition in distribution comparing zinc-limited vs. zinc-replete cells. A higher proportion of *MSC2*^*ATG2*^*-lacZ* mRNA was associated with polysomes in zinc-limited cells than in replete cells. These data are consistent with increased translation of the *MSC2*^*ATG2*^*-lacZ* mRNA in zinc-limited vs. replete cells. The control *CMD1* mRNA showed little such shift indicating that greater association with polysomes is not a general feature of mRNA in zinc-limited cells. These data also suggest that *CMD1* mRNA is efficiently translated and competes well for a potentially limiting number of ribosomes in zinc-limited cells.

### A zinc-responsive shift in the transcription start site of the *MSC2*^*ATG2*^*-lacZ* reporter gene

**Fig 4:**
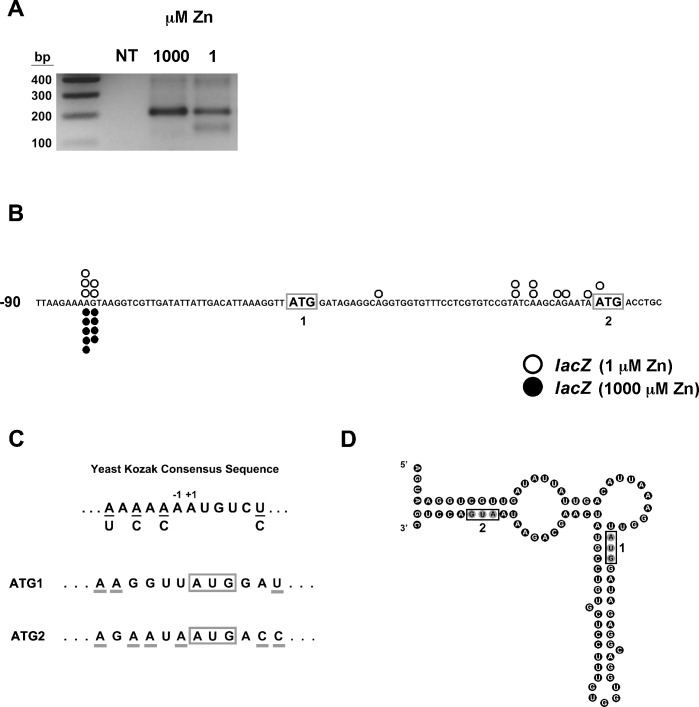
Zinc status affects transcription start site location in *MSC2*^ATG2^-*lacZ*. A) 5’ RLM-RACE products from zinc-replete (LZM + 1000 μM ZnCl_2_) or zinc-limited (LZM + 1 μM ZnCl_2_) cells expressing *MSC2*^ATG2^-*lacZ* were resolved on a 2% agarose gel and stained with ethidium bromide. NT indicates a no-template PCR control and lane 1 shows DNA size markers. B) *MSC2*^ATG2^*-lacZ* transcription start sites in zinc-limited (LZM + 1 μM ZnCl_2_) and replete (LZM + 1000 μM ZnCl_2_) cells. 5’ RLM-RACE fragments (fourteen from zinc-limited and nine from replete cell mRNA) were independently cloned and sequenced. The *open* circles indicate the start sites mapped in zinc-limited cells and the *filled* circles indicate zinc-replete start sites. ATG1 and ATG2 are *boxed* and in *bold*. C) Comparison of sequence context around ATG1 and ATG2 of *MSC2* relative to the yeast Kozak consensus sequence. The nucleotides flanking ATG1 and ATG2 that match the consensus are *underlined*. D) Mfold prediction of *MSC2*^ATG2^*-lacZ* long transcript mRNA folding. The predicated secondary structure has a Gibbs Free Energy (initial ***Δ***G) of -24 kcal/mol. AUG1 and AUG2 are *boxed*.

To explore potential mechanisms of translational control, we first determined the 5’ ends of *MSC2*^ATG2^-*lacZ* mRNAs. We used 5’ RLM-RACE to map the transcription start sites of ^7m^G-capped mRNA; this method allows for the specific PCR amplification of the 5’ends of mRNA. Total RNA was extracted from wild-type cells expressing *MSC2*^ATG2^*-lacZ* reporter after growth under zinc-limiting and replete conditions and used as a template for 5’ RLM-RACE and gene-specific primers were used in nested PCR reactions. When the PCR products were resolved by agarose gel electrophoresis, we noted a difference in *MSC2*^ATG2^-*lacZ* transcription start sites between the two conditions of zinc status (**Fig 4A**). *MSC2*^ATG2^-*lacZ* mRNA produced only longer 5’ RLM-RACE products when isolated from zinc-replete cells. DNA sequencing of independently cloned 5’ RLM-RACE fragments revealed transcripts starting at two adjacent sites located 33–34 nucleotides upstream of ATG1 (**Fig 4B**). 5’ RLM-RACE products from zinc-limited cells were resolved by agarose gel electrophoresis into two bands differing in size by ~60 bp. The longer, more abundant product co-migrated with the 5’ RLM-RACE band from zinc-replete cells and DNA sequencing of independently cloned fragments indicated that these mRNAs also initiated 33–34 nucleotides upstream of ATG1 (**Fig 4B**). The shorter 5’ RLM-RACE products from zinc-limited cell mRNA were also cloned and sequenced and several transcription start sites immediately upstream of ATG2 were identified. These results indicate that selection of transcription start sites for *MSC2*^ATG2^-*lacZ* is affected by zinc status.

The 5’ RLM-RACE results suggested that one possible explanation for the effects of zinc on β-galactosidase activity is that translation of the longer open reading frame starting from ATG1 could for some reason be less efficient. For example, the additional 16 codons encoded in the long transcript might be poorly translated and lead to less lacZ protein synthesis. Arguing against this model, comparison of ATG1 and ATG2 to the yeast Kozak consensus sequence [24] indicated that ATG1 is a very poor match to the consensus while ATG2 is a much better match **(Fig 4C)**. This observation suggests that ATG1 is not used as the primary site of translation initiation. An alternative mechanism was suggested by analysis of the potential folding of the 5’ ends of *MSC2*^ATG2^*-lacZ* mRNA. As shown in **Fig 4D**, the 5’ end of the long *MSC2*^ATG2^*-lacZ* transcripts that begin upstream of ATG1 can potentially fold into a relatively stable secondary structure. The predicted folded structure of these long transcripts has a Gibbs free energy (initial *Δ*G) of -24 kcal/mol and both ATG1 and ATG2 are included within the folded structure. In contrast, the 5’-UTRs of the shorter transcripts that start upstream of ATG2 in zinc-limited cells do not appear to have any stable folded structure (data not shown). Thus, the long transcripts that initiate upstream of ATG1 may be poorly translated because of their stable secondary structure and only the short transcripts initiating just upstream of ATG2 that are produced in zinc-limited cells are efficiently translated.

**Fig 5:**
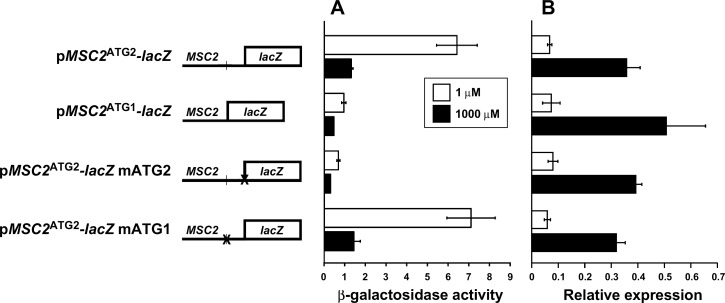
Assessing the roles of ATG1 and ATG2 in *MSC2-lacZ* expression. A) **β**-galactosidase activity was measured in wild-type (DY150) cells expressing *MSC2*^ATG2^*-lacZ*, *MSC2*^ATG1^*-lacZ*, *MSC2*^ATG2^*-lacZ* mATG2, *or MSC2*^ATG2^*-lacZ* mATG1 reporters and grown under zinc-limiting (LZM + 1 μM ZnCl_2_) or replete (LZM + 1000 μM ZnCl_2_) conditions. B) The *lacZ* mRNA abundance was also determined by quantitative RT-PCR. *lacZ* abundance was normalized to the average abundance of three control transcripts (18S rRNA, *TAF10*, and *ACT1*). The results are the means ± S.D. for three independent cultures for each condition.

To assess the relative roles of ATG1 and ATG2 in *MSC2*^ATG2^*-lacZ* translation, we generated a reporter that contains the *MSC2* promoter and the ATG1 initiation codon fused directly to *lacZ* (*MSC2*^ATG1^*-lacZ*) **(Fig 5**). Using this construct, we could test the translation efficiency of ATG1 specifically in zinc-limited vs. replete cells. In addition, two *MSC2*^ATG2^*-lacZ* mutant alleles were also generated in which either ATG1 (*MSC2*^ATG2^*-lacZ* mATG1) or ATG2 (*MSC2*^ATG2^*-lacZ* mATG2) were mutated to AAG, thereby rendering those sites incapable of translation initiation. Both of these alleles have predicted structures identical to *MSC2*^ATG2^*-lacZ* with only slightly altered *Δ*G values (-23 kcal/mol). Using β-galactosidase assays, we compared the activity of these four different constructs expressed in wild-type cells. Direct fusion of ATG1 to *lacZ* in *MSC2*^*ATG1*^*-lacZ* resulted in little β-galactosidase activity regardless of zinc status (**Fig 5A**). Mutation of ATG2 in *MSC2*^*ATG2*^*-lacZ* also abolished almost all β-galactosidase activity while mutation of ATG1 had no effect on zinc-responsive activity. When assayed by quantitative RT-PCR, *MSC2*^*ATG1*^*-lacZ* and the mutant constructs showed the same effects of zinc status on mRNA levels as *MSC2*^*ATG2*^*-lacZ*, indicating that the differences observed among the constructs were not due to differences in mRNA levels (**Fig 5B**). These results indicate that ATG1 is indeed a poor initiation codon in the context of these mRNA and translation initiation relies primarily on ATG2. Notably, the lack of an effect of mutating ATG1 in *MSC2*^ATG2^-*lacZ* demonstrates that ATG1 and the additional N-terminal codons do not have an inhibitory influence on translation of the mRNA. Therefore, the differential folding of the 5’-UTR is likely to be responsible for the zinc-responsive expression of this reporter.

**Fig 6:**
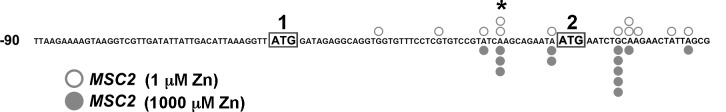
5’ RLM-RACE analysis of chromosomal *MSC2* transcription start sites. Mapping results of chromosomal *MSC2* transcription start sites in zinc-limited (LZM + 1 μM ZnCl_2_) and replete (LZM + 1000 μM ZnCl_2_) cells. 5’ RLM-RACE fragments (twelve from zinc-limited and thirteen from replete cell mRNA) were independently cloned and sequenced. The *open* circles indicate zinc-limited start sites and the *gray* circles indicate zinc-replete start sites. ATG1 and ATG2 are *boxed* and in *bold*. The *asterisk* marks the major transcription start site for *MSC2* mapped using mRNA from zinc-replete cells grown in rich media [25].

To investigate whether these effects alter expression of the chromosomal *MSC2* gene, we also assessed translation of *MSC2* mRNA by polysome profiling analysis in zinc-limited and replete cells. Similar to the *CMD1* control, there was little change in the distribution of *MSC2* mRNA across the gradients in response to zinc status **(Fig 3**). This result suggested that the chromosomal *MSC2* gene was not subject to the same changes in transcription start sites observed for *MSC2*^*ATG2*^*-lacZ*. Consistent with this hypothesis, when transcription start sites were mapped for chromosomal *MSC2* using 5’ RLM-RACE, no major shifts were observed (**Fig 6**). For chromosomal *MSC2*, all of the start sites mapped were in the vicinity of ATG2 and none were identified upstream of ATG1. Furthermore, these sites were near the major transcription start site mapped for *MSC2* using zinc-replete cells grown in rich media [25]. Therefore, the alterations in transcription start site were specific to the *MSC2*^*ATG2*^*-lacZ* reporter gene and not observed for the native gene. Notably, this effect is not due to the episomal status of the reporter; similar effects of zinc status on β-galactosidase activity were observed with the *MSC2*^*ATG2*^*-lacZ* reporter gene integrated into the genome (data not shown). Thus, we conclude that the sequence context surrounding the promoter in the *MSC2*^ATG2^*-lacZ* reporter gene plays some role in the altered transcription start site effects.

### One potential mechanism for altered start site selection in zinc-limited cells

**Fig 7:**
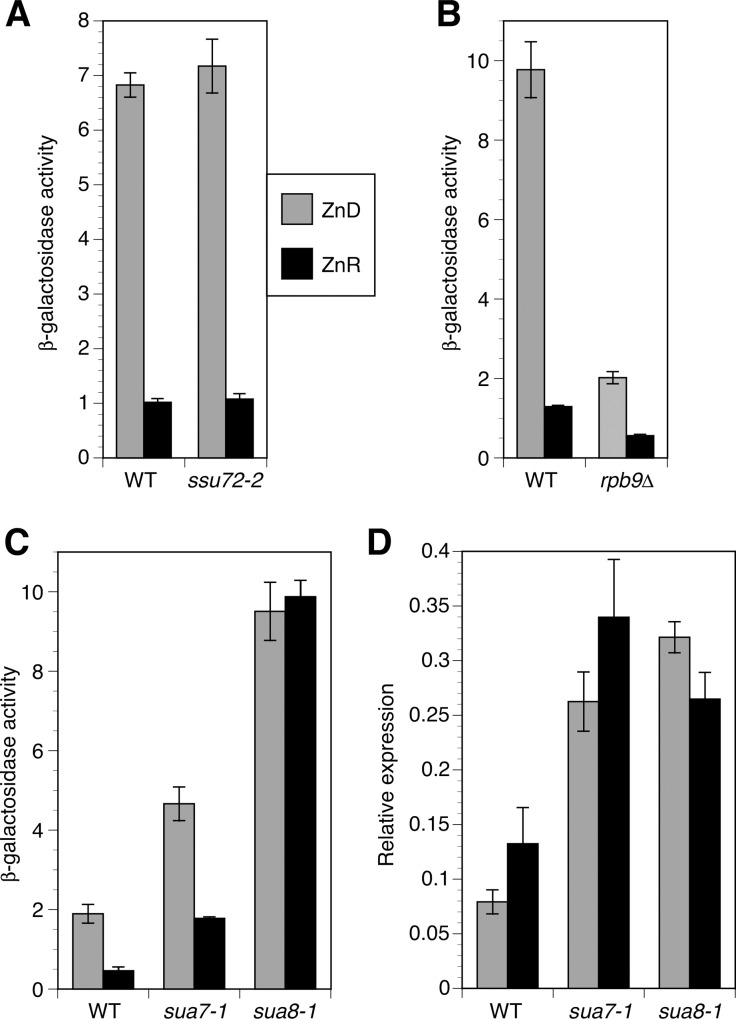
Mutations known to alter start site selection increase expression of the *MSC2*^ATG2^*-lacZ* reporter. Panels A-C) **β**-galactosidase activity was measured in cells of the indicated genotypes expressing the *MSC2*^ATG2^*-lacZ* reporter and grown under zinc-limiting (LZM + 1 μM ZnCl_2_) or replete (LZM + 1000 μM ZnCl_2_) conditions. It should be noted that the wild-type strains used in each panel are different from each other but isogenic to the mutant strain(s) with which they are compared. D) The *lacZ* mRNA abundance in the cells from panel C was also determined by quantitative RT-PCR. *lacZ* abundance was normalized to the average abundance of three control transcripts (18S rRNA, *TAF10*, and *ACT1*). The results are the means ± S.D. for three independent cultures for each condition.

Many components of the transcriptional machinery are involved in transcription start site selection and several of these are zinc-binding proteins. We hypothesized that the shift in transcriptional start sites observed for *MSC2*^*ATG2*^*-lacZ* could be due to loss of zinc binding by one or more of these factors and an accompanying alteration in start site selection. Zinc-binding proteins known to influence start site selection include the RNA polymerase II subunits Rpo21 (Sua8) and Rpb9, TFIIB (Sua7), and the TFIIB-associated protein Ssu72 [9–12]. To test this model, we assayed *MSC2*^*ATG2*^*-lacZ* expression in strains with mutations that disrupted function of these proteins that were previously shown to alter transcription initiation sites. As shown in **Fig 7A**, the *ssu72-2* hypomorphic mutation had no effect on lacZ activity in zinc-limited and replete cells. An *rpb9Δ* mutation reduced expression of the reporter in both zinc-limited and replete cells but relative zinc responsiveness was largely unaffected (**Fig 7B**). In contrast, mutations affecting both TFIIB (*sua7-1*) and Rpo21 (*sua8-1*) increased lacZ activity in zinc-limited and replete cells and also rendered overall expression less zinc responsive (**Fig 7C**). While zinc supplementation reduced lacZ activity in *sua7-1* by ~60%, it had no effect on lacZ activity in the *sua8-1* strain. To determine the relationship of these changes in lacZ activity to mRNA levels, we measured *lacZ* mRNA in these cells by quantitative RT-PCR. The wild-type strain showed an ~2-fold decrease in *lacZ* mRNA when grown in the zinc-limited medium vs. zinc-replete conditions (**Fig 7D**) that was qualitatively similar to what we observed in other wild-type strains (e.g. **Fig 1D**). In contrast, the *sua7-1* and *sua8-1* strains showed constitutively elevated mRNA levels. The observation that the 2-fold increase in lacZ mRNA level in zinc-replete mutant cells is accompanied by a 4-fold (*sua7-1*) or 20-fold (*sua8-1*) increase in lacZ activity is consistent with a shift in transcription initiation to sites proximal to ATG2. Thus, these data are consistent with decreased metallation and altered function of Rpo21 and/or TFIIB in zinc-limited cells causing a shift in transcription initiation from nonfunctional sites upstream of ATG1 to the functional sites near ATG2. The increased accumulation of *lacZ* mRNA in the mutant strains could be explained if the shorter translated mRNA are more stable than the longer mRNA that are not translated.

### Genome-wide effects of zinc status on intragenic transcription initiation

**Fig 8:**
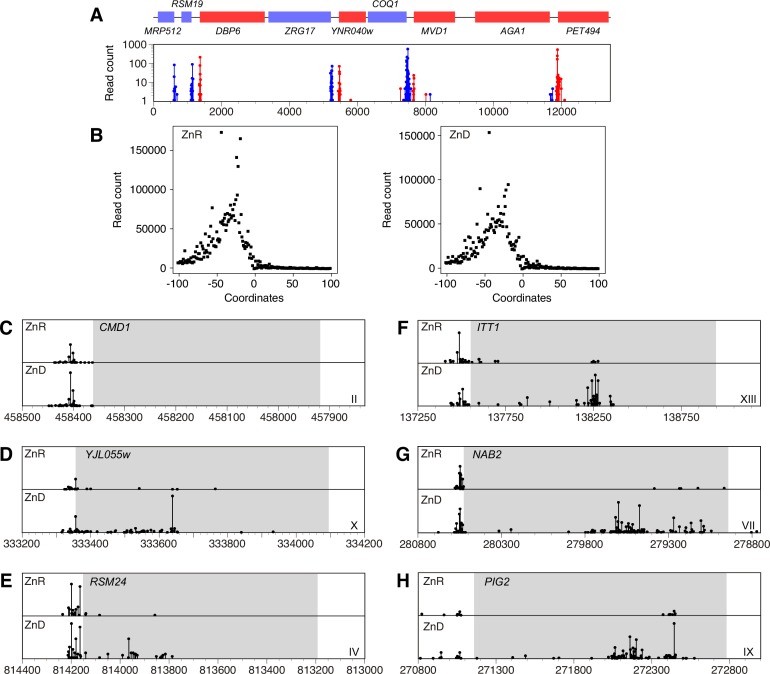
Mapping transcription start sites by 5’ Deep-RACE. A) Transcription start sites mapped for an ~13 kb region of yeast chromosome XIV is shown. Genes indicated in red are transcribed from left to right while those in blue are transcribed from right to left. Independent sequencing reads, representing mRNA 5’ ends, are plotted with the same color scheme across the region. B) The transcription start sites mapped for all genes are plotted relative to the translation initiation codon of the open reading frame numbered as +1. Data obtained from zinc-replete and zinc-limited cells are plotted. C-H) Transcription start sites for the indicated genes are plotted relative to the open reading frame shown in gray. The chromosome of each gene is indicated by the Roman numeral and the coordinates represent the location on the corresponding chromosomes.

The effects of zinc status on *MSC2*^ATG2^*-lacZ* transcription initiation suggested that similar changes might be affecting the expression of other genes. To test this hypothesis, the 5’RLM-RACE method was adapted to the genome-wide analysis of transcription start sites using 5’ Deep-RACE [21]. With this method, the 5’ends of the mRNA of all expressed genes in zinc-limited and replete cells were specifically amplified and sequenced by Illumina sequencing. A sample region of chromosome XIV is shown in **Fig 8A** to illustrate the data obtained and the complete normalized dataset is provided in **S1 Table**. When all genes were aligned by their initiation codons, the distribution of transcription start sites relative to the ATG codon was similar in zinc-limited vs. zinc-replete mRNAs (**Fig 8B**). Peak 5’UTR lengths were ~30 nucleotides regardless of zinc status. This result suggests that transcription start sites in most of the genome are largely unaffected and the start sites in the *CMD1* gene are shown in **Fig 8C** as one example. However, while the majority of genes in the genome showed little change in transcription start site selection, manual inspection of the data identified a significant fraction (165 of the ~6000 genes total, 2.8%) [**S2 Table**] that displayed altered initiation sites under zinc-limiting conditions. The effects of zinc status on transcription initiation were highly variable among these genes but most affected genes showed increased transcription initiation at sites within their open reading frames. For example, a broad distribution of intragenic start sites were observed in the 5’ half of the *YJL055W* and *RSM24* coding regions (**Fig 8D and 8E**) while more promoter-distal intragenic sites was observed for *ITT1*, *NAB2*, and *PIG2* (**Fig 8F–8H**). These results suggest that the *MSC2*^ATG2^*-lacZ* may be serving as an indicator of broader effects of zinc status on transcription of the genome. Gene Ontology analysis of the affected genes indicated that they were diverse and not biased with respect to gene groupings categorized by protein/enzymatic function, cellular process, or spatial localization. This observation suggests that the changes in transcription start sites were not due to altered activity of specific regulatory transcription factors but were instead due to effects of zinc deficiency on more general factors such as chromatin structure or basal transcription machinery components.

## Discussion

In this study, we set out to determine whether expression of the *MSC2* gene was regulated in response to zinc status. This was motivated by our interest in an integrated understanding of the regulation of zinc homeostasis in the early secretory pathway. The Msc2 and Zrg17 proteins of *S*. *cerevisiae* are both members of the cation diffusion facilitator family of metal transporters [4]. These two proteins form a heteromeric complex in the ER membrane to mediate zinc transport into this organelle. While we previously showed that the *ZRG17* gene is a direct target of the Zap1 transcription factor and its expression is induced many fold in zinc-limited cells [1], we knew nothing about *MSC2* regulation.

Our previous microarray analyses of zinc-responsive gene expression failed to detect changes in *MSC2* mRNA in response to zinc [6,7]. However, it is well recognized that DNA microarrays can be relatively insensitive and often fail to detect small but biologically significant changes in gene expression [26]. Using two more sensitive independent methods (i.e. S1 nuclease protection assay and quantitative RT-PCR), we found that *MSC2* mRNA levels consistently increased by ~1.5 fold in zinc-limited cells relative to replete cells. Thus, it appears that both *ZRG17* and *MSC2* genes are under zinc-responsive transcriptional control. Because both proteins are required for transport function, simultaneous induction of these proteins likely facilitates more efficient zinc transport into the lumen of the ER. We cannot yet say whether *MSC2*, like *ZRG17*, is also regulated by Zap1.

Fusing a 500 bp *MSC2* promoter fragment to *lacZ* failed to confer normal *MSC2* regulation on the reporter gene. Surprisingly, while *MSC2*^*ATG2*^*-lacZ* mRNA decreased 4-fold in zinc-limited cells, the abundance of its protein product, β-galactosidase, increased ~7 fold. This difference suggested that lacZ activity was under some sort of post-transcriptional influence and we therefore investigated the mechanism responsible for this effect. One possible explanation was that lacZ protein was degraded more quickly in zinc-replete cells and therefore accumulated to higher levels in zinc-limited cells. However, when we tested lacZ stability by cycloheximide chase analysis we found the opposite result; lacZ is more unstable in zinc-limited cells. Therefore, differences in lacZ degradation are not responsible for the observed effects of zinc status.

To assess possible mechanisms of translational effects, we mapped the 5’ ends of *MSC2*^*ATG2*^*-lacZ* mRNA in zinc-limited and replete cells. We discovered that zinc status affected the sites of transcription initiation in this construct and our subsequent experiments led to the conclusion that these changes in transcription start site were likely responsible for the disparity between the mRNA and activity changes. Specifically, our mutational analysis indicated that ATG1 was a poor initiator codon and the lacZ protein was being translated starting from ATG2. Thus, the mRNAs from zinc-replete cells have 5’ UTRs of 80–81 nucleotides while the short transcripts mapped in mRNA from zinc-limited cells have 5’ UTRs that range from 1–36 nucleotides. The long-form 5’ UTRs are predicted to fold into a stem-loop secondary structure with a *Δ*G value of -24 kcal/mol. This stability is likely sufficient to block translation initiation given that 5’ UTR stem-loop structures with *Δ*G of <-10 kcal/mol could block translation initiation in yeast by 90% or more [27,28]. It is also possible that the long form of the *MSC2*^*ATG2*^*-lacZ* 5’ UTR is bound by unknown protein factors and this prevents translation initiation, although we have no evidence for the existence of such factors.

Our model also proposes that the short-form 5’ UTR mRNA found in zinc-limited cells are more efficiently translated than their longer counterparts. This prediction was supported by the observation that *MSC2*^*ATG2*^*-lacZ* mRNA was more abundant on polysomes in zinc-limited cells, where the short forms are produced, than in zinc-replete cells where only the long forms are generated. The minimum number of nucleotides needed to be present in a yeast 5’ UTR for translation to occur is not precisely known and we recognize that some of the shortest transcripts we identified may not be functional. However, the majority of yeast mRNAs have 5’UTRs under 50 nucleotides and, remarkably, many have 5’ UTRs smaller than 5 nucleotides [29,30].

What mechanism is responsible for the zinc-responsive shift in the sites of transcription initiation? While the answer to this question is not yet known, we are currently considering a few possibilities. One potential hypothesis is based on the fact that several basal transcription factors bind zinc and require the metal for their function. These include subunits of RNA polymerase II (Rpo21/Sua8, Rpb9), TFIIB (Sua7), and the TFIIB-associated factor Ssu72. Notably, mutations affecting all of these cofactors have been found to alter transcription start site selection [9–12]. We reasoned that under zinc deficiency, some of these factors are not fully metallated and this would alter transcription start site selection to some degree. If correct, mutations that disrupt the function of the relevant factor may cause constitutive use of the downstream transcriptional start sites we observed in zinc-deficient cells. We assayed *MSC2*^*ATG2*^*-lacZ* reporter expression in several such mutants and were intrigued to find that point mutations in the Rpo21 large subunit of RNA polymerase II (*sua8-1*) and in TFIIB-encoding *SUA7* resulted in high β-galactosidase activity from the *MSC2*^*ATG2*^*-lacZ* reporter regardless of zinc status. While these results do not demonstrate that impaired zinc binding by Pol II and/or TFIIB is responsible for the shift in the sites of transcription initiation we observed, they are consistent with that model.

A second plausible hypothesis is that the transcription start site changes reflect activation of cryptic promoter elements within the *MSC2*^*ATG2*^*-lacZ* reporter in zinc-limited cells due to alterations in chromatin. Histone deacetylases such as Rpd3 and Hda1 play important roles in stabilizing chromatin structure and repressing transcription of cryptic as well as productive promoters [31–33]. Rpd3 and Hda1 are members of the class I and class II HDAC proteins, respectively, and both are dependent on zinc binding for their function [34]. Therefore, decreased HDAC activity in zinc-limited cells as a result of undermetallation, and the concomitant disruption of chromatin structure, could conceivably lead to increased activity of cryptic promoter elements in the *MSC2*^*ATG2*^*-lacZ* fusion. The promoter-distal effects observed for genes like *ITT1*, *NAB2*, and *PIG2* are consistent with cryptic promoter activation (**Fig 8F–8H**). Ongoing studies are addressing the possible mechanisms affecting transcription initiation in zinc-limited cells. We believe that multiple mechanisms are likely and these may affect different genes to varying degrees.

Whatever the mechanism, we predicted that at least some chromosomal genes in the yeast genome are also affected by the process altering *MSC2*^*ATG2*^*-lacZ* transcription initiation. Clearly, major disruption of transcription start site selection or cryptic promoter repression would have lethal consequences to the cell. However, changes in this process for a few genes could be tolerated to some degree and this might contribute to the poor growth we observe for zinc-limited cells. For example, it was recently shown that mutations in *RPD9*, which alters transcription start site selection [12], induces a stress to protein homeostasis, slows growth, and shortens cellular lifespan [35]. While we found no evidence that the *rpd9Δ* mutation alters *MSC2*^*ATG2*^*-lacZ* transcription initiation, it is likely that other factors that cause similar changes in zinc-limited cells would also disrupt protein homeostasis and slow growth. We noted previously that zinc deficiency disrupts protein homeostasis [36]. While we hypothesized at the time that this stress arose from the accumulation of unfolded zinc apoproteins, a disruption in the specificity of transcription initiation could also be a major contributing source of unfolded proteins. In this scenario, the *MSC2*^*ATG2*^*-lacZ* reporter may be serving as a useful harbinger of a broader problem that the cell is experiencing. In contrast, we recognize the possibility that some of the transcription start site changes observed may be regulatory in purpose rather than due to a disruption in normal function. In fact, we have discovered two bona fide regulatory effects among the ~170 genes we identified as having altered transcription start sites and our characterization of those zinc-responsive regulatory switches is underway.

Many previous repeats have mapped the 5’ends of yeast mRNA genome wide using various means such as 5’SAGE and RNA-Seq [25,37–39]. These mapping experiments were all performed using mRNA from cells grown in rich media. For our analysis, the cells were grown in a minimal medium and thus, we provide here the first comprehensive analysis of transcription start sites for cells grown in other conditions than previously reported. Because gene expression changes greatly between rich and minimal media, our analysis of transcription start sites in zinc-replete cells will be of value to researchers studying yeast gene expression in minimal media conditions.

Finally, the observation that the *MSC2*^*ATG2*^*-lacZ* reporter behaves differently than the chromosomal gene highlights the often ignored fact that reporter gene fusions must be used with great caution when they are employed as assays for effects on endogenous, chromosomally-encoded genes. Literally thousands of published papers have relied on promoter-*lacZ* fusions and analogous reporter genes (e.g. luciferase, β-glucuronidase) and our study serves as a remarkable cautionary tale to remind us that reporter gene constructs do not always accurately reflect normal regulatory effects. Removal of a gene’s promoter from its normal context, and insertion of new flanking vector and reporter gene sequences can alter promoter activity. A handful of studies have noted this problem but we suspect that many artifactual results have gone unrecognized [40,41].

## Supporting Information

S1 Table Transcription start sites in zinc-replete and deficient yeast. Transcription start sites in the yeast genome mapped in zinc-replete (ZnR, 8 x 10^6^ total tags) and zinc-deficient (ZnD, 6 x 10^6^ total tags) cells. Coordinates refer to the nucleotide position of the mRNA 5’ end on the corresponding chromosome and the number of reads for each site are normalized to 10^7^ tags.
(XLSX)

S2 Table Genes identified with transcription start sites that are altered by changes in zinc status. (XLSX)
